# Zileuton, 5-Lipoxygenase Inhibitor, Acts as a Chemopreventive Agent in Intestinal Polyposis, by Modulating Polyp and Systemic Inflammation

**DOI:** 10.1371/journal.pone.0121402

**Published:** 2015-03-06

**Authors:** Elias Gounaris, Michael J. Heiferman, Jeffrey R. Heiferman, Manisha Shrivastav, Dominic Vitello, Nichole R. Blatner, Lawrence M. Knab, Joseph D. Phillips, Eric C. Cheon, Paul J. Grippo, Khashayarsha Khazaie, Hidayatullah G. Munshi, David J. Bentrem

**Affiliations:** 1 Department of Surgery, Northwestern University Feinberg School of Medicine, Chicago, Illinois, United States of America; 2 Robert H. Lurie Comprehensive Cancer Center, Northwestern University Feinberg School of Medicine, Chicago, Illinois, United States of America; 3 Jesse Brown Veterans Affairs Medical Center, Chicago, Illinois, United States of America; National Health Research Institutes, TAIWAN

## Abstract

**Purpose:**

Leukotrienes and prostaglandins, products of arachidonic acid metabolism, sustain both systemic and lesion-localized inflammation. Tumor-associated Inflammation can also contribute to the pathogenesis of colon cancer. Patients with inflammatory bowel disease (IBD) have increased risk of developing colon cancer. The levels of 5-lipoxygenase (5-LO), the key enzyme for leukotrienes production, are increased in colon cancer specimens and colonic dysplastic lesions. Here we report that Zileuton, a specific 5-LO inhibitor, can prevent polyp formation by efficiently reducing the tumor-associated and systemic inflammation in APC^Δ468^ mice.

**Experimental Design:**

In the current study, we inhibited 5-LO by dietary administration of Zileuton in the APC^Δ468^ mouse model of polyposis and analyzed the effect of *in vivo* 5-LO inhibition on tumor-associated and systemic inflammation.

**Results:**

Zileuton-fed mice developed fewer polyps and displayed marked reduction in systemic and polyp-associated inflammation. Pro-inflammatory cytokines and pro-inflammatory innate and adaptive immunity cells were reduced both in the lesions and systemically. As part of tumor-associated inflammation Leukotriene B4 (LTB4), product of 5-LO activity, is increased focally in human dysplastic lesions. The 5-LO enzymatic activity was reduced in the serum of Zileuton treated polyposis mice.

**Conclusions:**

This study demonstrates that dietary administration of 5-LO specific inhibitor in the polyposis mouse model decreases polyp burden, and suggests that Zileuton may be a potential chemo-preventive agent in patients that are high-risk of developing colon cancer.

## Introduction

Increased chronic colitis predispose patients to colorectal cancer (CRC) and colitis-associated cancer (CAC) [1–5]. The tumor microenvironment is characterized by high leukocyte infiltration that vary in size, distribution and composition [6–8]. Tumor-associated macrophages (TAMs), mast cells (MCs), dendritic cells (DCs), natural killer cells (NKs), neutrophils, eosinophils and lymphocytes infiltrate tumors [9–13]. These inflammatory cells produce and secrete pro-inflammatory cytokines, and interferons. Most of these pro-inflammatory cells display high levels of cyclooxygenase-2 (COX-2), 5-lipoxygenase (5-LO) [14] [15] and phospholipase A2. Prostaglandins and leukotrienes, the end products of arachidonic acid, which are produced by the pathways of COX-2 and 5-LO respectively, are known to sustain the inflammatory reaction both in the lesions and systemically.

Arachidonic acid (AA) is liberated from membrane phospholipids by phospholipase A2 and then metabolized into Leukotriene B4 (LTB4) by the 5-LO/5-LO activating protein (FLAP) complex. FLAP is predominantly located in the nuclear envelope of mast cells, macrophages, and myeloid suppressor derived cells [16,17]. Significantly, 5-LO is associated with CRC progression. We have previously shown that 5-LO antigen is up-regulated in patients with polyps and colon cancer[15]. In polyposis mouse model, we also demonstrated that loss of 5-LO was protective [18]. In the present study, we examined the effect of pharmacological inhibition of 5-LO with Zileuton in mice on intestinal polyposis. Zileuton (Zyflo) is an oral 5-LO inhibitor approved by the FDA for the treatment of patients with asthma. We demonstrated that APC^Δ468/+^ mice fed with chow containing Zileuton for 12 weeks developed fewer polyps and concurrently showed lower serum LTB4 concentrations. This outcome verified the inhibition of 5-LO by Zileuton. As a result of the treatment we observed a decrease in the levels of inflammatory reaction. The levels of serum pro-inflammatory cytokines, the mature monocytes, and the pro-inflammatory T-regs were significantly reduced after chemo-preventive treatment. Similarly tumor-infiltrated mast cells, macrophages, MDSCs and pro-inflammatory T-regs were significantly reduced focally in the polyps after the Zileuton chemo-preventive treatment. Overall, our findings suggest that Zileuton could potentially be used as a chemo-preventive agent in patients that are high-risk of developing colon cancer.

## Materials and Methods

### Patient samples

Informed consent was obtained from all participants. The Scientific Review Committee of Northwestern University approved IRB protocols. All patients had histologic diagnosis of colon cancer and had not received any prior therapy. All tumors were tested for microsatellite instability (MSI) resulting from abnormalities in DNA mismatch repair genes (MLHa, MutL and MutS). MSI-positive tumors were excluded from this study.

### Animals

Four-week-old APC^Δ468^ mice with a genetic background of C57BL/B6 were given AIN93G chow either with or without 1200 mg/kg[19] Zileuton homogenized into pellets (*n* = 8 mice per group). Mice were sacrificed at four months of age. Zileuton was acquired from Cornerstone Therapeutics Inc. (Cary, NC). Test Diet (Richmond, Indiana) homogenously incorporated Zileuton into the AIN93G chow that was previously purchased from Research Diets, Inc. (New Brunswick, NJ).

All animal work was approved and conducted under the guidelines of Northwestern University’s Animal Care and Use Committee.

### Histology and immunofluorescence

Mouse small intestine and colon were fillet-opened, Swiss-rolled, either frozen or fixed in formalin, processed in paraffin, and sectioned at 5μm. Slides were then de-paraffinized, hydrated, stained with H&E, and assessed by a pathologist. The tissue contained intra-mucosal tubular adenoma and tubular adenocarcinoma arising from tubular adenoma. For paraffin-embedded sections, heat-mediated antigen retrieval was performed using 1X Target Retrieval Solution (DAKO) while frozen sections were fixed in ice-cold methanol for 15 minutes. All sections were blocked with Cyto Q Background Buster (Innovex Biosciences) containing 1:200 Fc Block and then incubated overnight at 4°C with purified rat anti-mouse Gr-1 (Caltag Laboratories), F4/80 (BioLegend), CD4 (BD Bioscience), or mouse anti-mouse Foxp3 (eBioscience) antibodies. Sections were washed with PBS and incubated in the dark at room temperature with anti-rat Alexa Fluor 488 or anti-mouse Alexa Fluor 594 (Invitrogen) for two hours. Sections were washed in PBS, incubated for 20 minutes with DAPI (Invitrogen), washed in PBS, and mounted with anti-fade mounting medium containing DAPI. Fluorescent images were collected with a TissueGnostics Cell Analyzer, and frequencies of cells were analyzed using Image J software.

### TUNEL and BrdU staining

*TUNEL staining*: The ApopTag Peroxidase *in situ* apoptosis detection kit (Chemicon International) was used according to the manufacturer’s instructions.

*BrdU staining*: Two hours prior to sacrifice, mice were injected with 20 mg/kg BrdU (Sigma). Paraffin embedded sections were incubated overnight at 4°C with a BrdU antibody (Chemicon/Millipore). Sections were treated with HRP-linked secondary antibody (DAKO) and developed with DAB reagent. Light microscopic images were collected with a Leica DCC camera. Frequencies of cells were analyzed using ImageJ software.

### FACS

Cells were incubated with Fc Block (BD Bioscience) for 20 minutes. Live/Dead Violet Dead Cell Stain kit (Invitrogen) was used along with the murine antibodies: CD3-FITC, CD4-APC, CD8-PO, Foxp3-PE-Cy7, CD11b-PE, F4/80-Fitc (BD Biosciences). Flow cytometry was performed using the FACS Canto II instrument (BD Biosciences). Data was analyzed using the Flow Jo software (Tree Star).

### NIRF endoscopy

Detailed protocol of near infrared fluorescence endoscopy has been previously published [20]. Briefly, mice were injected with 2 nmol of ProSense 680, 16 to 24 hours before the endoscope session. The mouse colons were washed with PBS prior to inserting BF-XP60, fiberscope (Olympus, Tokyo, Japan). The excitation light (680 nm) of the prototype endoscope was produced after spectral separation of the white light produced from a Xe lamp. Collected images (300 · 300 pixels) were converted to time stacks and videos with the use of the NIH Image J software. Three regions of interest (ROI; 30 · 30 pixels) were selected throughout the stack and the mean fluorescence intensity (MFI) of each ROI was calculated with the Image J and plotted with the GraphPad Prism5 software.

### Anesthesia

Throughout the in vivo procedures, mice were anaesthetized with inhalation of 1.5% to 2% mixture of isoflurane in oxygen (1 L/h) according to the approved CCM protocol.

### ELISA

Mouse blood was collected into micro-centrifuge tubes and allowed to clot, centrifuged, and supernatant was collected. Multiplex ELISA was conducted on the mouse serum according to the manufacturer’s instructions (Millipore). Results were acquired with a Luminex 100 instrument and analyzed using xponent software (Luminex Corporation). Levels of LTB4 in mouse serum and human tissue lysates from tumor and adjacent normal tissue obtained during surgical resection was determined with the LTB4 Parameter Kit according to manufacturer’s instructions (R&D Systems).

### Statistical analysis

Prism 5 (GraphPad Software) was used for statistical analysis. ***P*** values were determined with one tailed unpaired *t*-tests.

## Results

### Inhibition of 5-LO suppresses polyp development

A group of 6 APC^Δ468/+^ mice were fed with chow AIN93G containing Zileuton (Zileuton; 1,200mg/kg). The treatment was started at the age of 4 weeks and continued until their euthanasia at the age of 16 weeks (Zileuton group). The number and the size of the polyps were originally calculated by optical observation of flayed small intestine and colon under a dissection microscope. Eight 16-week old APC^Δ468/+^ mice fed with Zileuton-free AIN93G chow were used as controls for the polyp development phenotype (control group).

**Fig 1:**
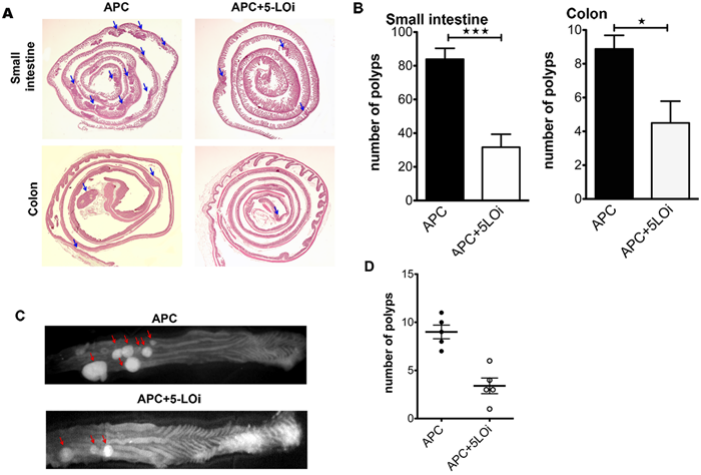
Zileuton is inhibiting the polyp formation in APCΔ468 colon and small intestine. A) Representative H&E stained 5μm Swiss rolls sections of small untreated intestine and colon of Zileuton treated and control APC^Δ468^ mice, blue arrows indicate polyps appearing in this sections. B) Cumulative bar graphs of the polyp number appearing in APC^Δ468^ mice small intestine (31.67±7.7 polyps in Zileuton group, n = 6, vs 83.88±6.5 polyps, n = 8, in the control group, p = 0.0002, closed bars) and colon (4.5±1.3 polyps in Zileuton group vs 8.9±0.8 polyps in control group, P = 0.0107, open bars, N = 6). C) Reflectance fluorescence of flayed opened colon, probe ProSense 680, representative whole mount of APC^Δ468^ colon of APC^Δ468^ Zileuton fed colon; red arrows polyps D) dot plot of the number of polyps; (3 closed dots) APC^Δ468^ untreated colon polyps, (3 open dots) APC^Δ468^ treated colon polyps. Statistics: unpaired one tailed t test.

Mice from the Zileuton group developed 2.6 times fewer polyps in the small intestine and half the number of polyps in the colon (Fig. 1A,B). We confirmed these findings through H&E staining of Swiss rolls of the small intestine and the colon. Representative H&E images of the small intestine and colon Swiss roles are in Fig. 1A). It should be noted that the typical H&E stained section is 5μm, which is a very small fraction of the width of a typical Swiss roll, which is 150 μm fold wide.

To verify the number of colon polyps, we injected five Zileuton and five control mice with 2nmoles of cathepsin activity NIRH probe ProSense 680 (Perkin Elmer, MA, USA), each 24 hours before euthanasia. The flayed open colons were imaged *in vivo* using reflectance fluorescence. We previously published that cathepsin activity is focally increased in the polyp region because of the focal increase in the number of myeloid cells in the microenvironment[20,21]. The NIRF cathepsin activity probe, like ProSense 680, serves as sensitive and specific surrogate marker for the detection of polyps and dysplastic lesions. Images of colon whole mounts revealed focal increase in emissions that correspond to polyps (Fig. 1C). The number of the colon polyps in the Zileuton-treated mice, as calculated with NIRF reflectance fluorescence was at least 2.5 fold less than the polyps in the control group (Fig. 1D). We had similar observation with reflectence fluorescence, in which the staining for cathepsin activity was *in vivo* and the observation was *ex vivo (* S1 Fig.).

### Zileuton reduces the proliferation rate of polyp cells in both small intestine and colon

**Fig 2:**
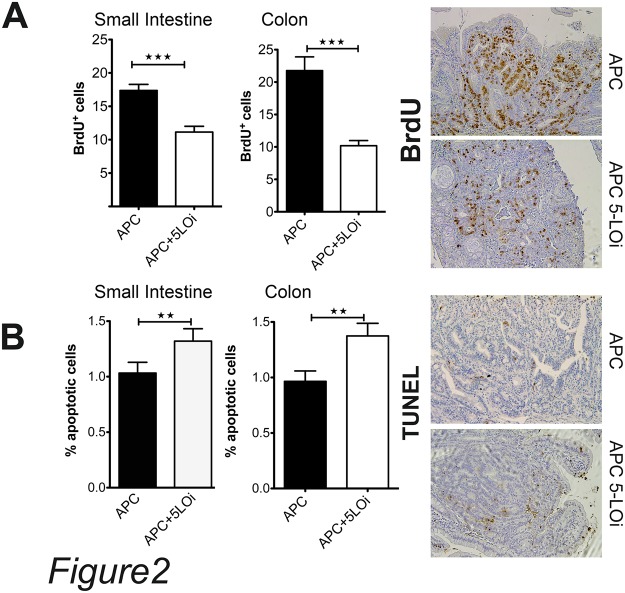
Zileuton treatment inhibits the proliferation rates of non epithelial cells in polyps, and increases the apoptosis rates in polyps. A) Polyp BrdU^+^ cells in small intestine and colon of Zileuton treated and control APC^Δ468^ mice. Black bars total BrdU^+^ in APC^Δ468^ polyps, open bars total BrdU^+^ in Zileuton APC^Δ468^ polyps (17.4±0.91 BrdU^+^ cells per high power field in the control group vs 11.13±0.87 BrdU^+^ cells in Zileuton fed group; P = 0.0001). B) Polyp apoptotic cells in small intestine and colon of Zileuton treated APC^Δ468^ polyps (open bars) and APC^Δ468^ polyps (black bars).One tailed unpaired t test.

Since Zileuton-treated mice developed fewer polyps than the control group, we injected the Zileuton-fed mice with BrdU to calculate the mitotic rate of the polyp cells. In the polyps of the small intestine, the number of BrdU^+^ cells was 30% lower in the Zileuton group compared to the mice of the control group, while in the colon polyps the frequency of the dividing cells was over 2-fold lower in the Zileuton group than the control group (Fig. 2A).

Furthermore, we observed a small but a significant increase in the number of apoptotic cells in the Zileuton-treated cells both in small intestine and in the colon (Fig. 2B). The reduced proliferation rate may significantly contribute to the reduction of polyposis in both the small intestine and colon of Zileuton-fed APC^Δ468^ mice.

### Pharmacological inhibition of 5-lipoxygenase modulates the systemic inflammation of APC^Δ468^ polyposis mice

At the age of 16 weeks APC^Δ468^ mice were cachectic on average weighing 30% less with 3 fold increased spleen weight compared with normal mice. Parmacological treatment of the APC^Δ468^ mice with Zileuton from the 4th week until the euthanasia reversed this phenotype. The Zileuton-treated cohort of mice had increased weight and their spleen was much smaller (S1 Fig.). We hypothesized that this change in the phenotype could be attributed to the change in the systemic inflammatory environment in the mice due to the Zileuton treatment.

**Fig 3:**
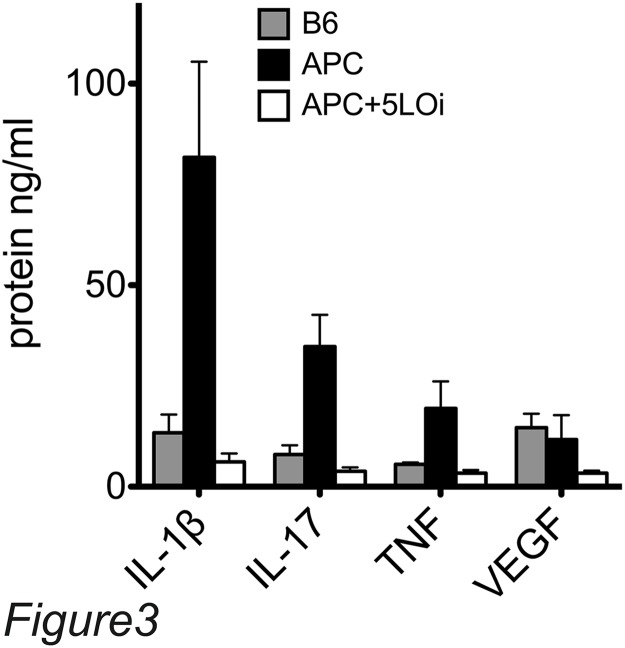
Zileuton treatment reduces the systemic inflammation in APC^Δ468/+^ mice. Serum multiplex ELISA; open bars APC^Δ468^Zileuton treated serum, black bars APC^Δ468^ serum, gray bars B6 serum.

A Multiplex ELISA revealed that APC^Δ468/+^ mice had increased systemic inflammation, displaying a Th-17 type of reaction, since the levels of serum IL-1β, IL-17, and TNF-α are very high in comparison to the control C5BL/6 mouse serum. The levels of cytokines fell to the level of B6 control mice when the APC^Δ468^ were fed Zileuton chow (Fig. 3).

**Fig 4:**
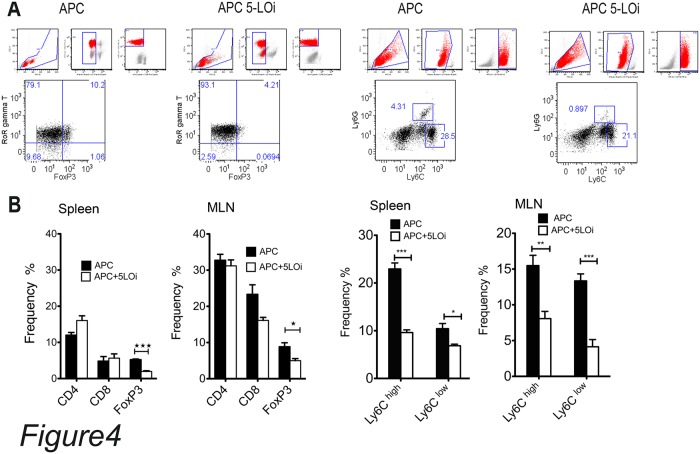
Frequency of lymphoid and myeloid cells isolated from APCΔ468Zileuton treated and APCΔ468 spleen and mesenteric lymph nodes (MLN). A) representative dot plots and the gating procedure of single cells suspencions isolated from APC^Δ468^ and APC^Δ468^Zileuton treated spleens. B) cummulative results of flow cytomentry of both spleen and MNL single cell suspencions. Black bars APC^Δ468^, and open bars APC^Δ468^Zileuton treated cellls. Flow cytometry analyzed with flow jo software, *One tailed unpaired* t test.

IL-17 is mainly produced by CD4+ T-cells and more specifically by T-regs that are FoxP3^+^ IL-17^+^ and display a pro-inflammatory phenotype. We analyzed the frequencies of these cell populations with flow cytometry using single cell suspention of spleen cells and mesenteric lymph node cells (Fig. 4A). The single cell suspention of these tissues were stained according to the materials and methods. The number of CD4^+^ FoxP3^+^ cells were decreased 3 fold in the spleen and 2 fold in the mesenteric lymph nodes (MLN) of the mice from the Zileuton-fed group. Furthermore, we observed 50% decrease in the FoxP3^+^ RORγt^+^ frequencies in the both the spleen and MNL of Zileuton treated mice. This may suggest that the systemic inflammatory reaction was attenuated due to reduction in the number of the pro-inflammatory Tregs.

To evaluate whether monocytes contributed to the reduction in inflammation in the Zileuton-fed mice, we analyzed single cell suspensions of spleens and MLNs with flow cytometry (Fig. 4B). We stained the cells with Ly6C, Ly6G(Gr1), F4/80 antibodies. The myeloid cells with the Ly6C^high^, Ly6G^high^ phenotype are immature monocytes distributed in lymphoid organs where they mature. A fraction of them are F4/80^high^ too. The Ly6C^low^, Ly6G^high^ type of cells are mature monocytes that sustain the inate inflammatory reaction in the tissue [22]. APC^Δ468^ spleen and MLN cells contain high frequency of Ly6C^high^, Ly6G^high^ and fairly high frequency of Ly6C^low^, Ly6G^high^ monocytes. The fequencies of Ly6C^high^, Ly6G^high^ and Ly6C^low^, Ly6G^high^ in Zileuton-treated spleens and MLNs are signifantly lower and this might explain the systemic reduction in the inflammatory reaction (Fig. 4).

### Zileuton treatment reduces the infiltrating pro-inflammatory cells in the polyps of APC^Δ468^ mice

It is well documented that tumor associated inflammation is required in the development of the early dysplastic lesions. The tumor cell survival is dependent of growth factors, like TNFα, IL1β, IL6 and VEGFα secreted by mast cells, macrophages and MDSCs. We have shown that Zileuton chemopreventive treatment reduced the systemic inflammation of the 16 weeks old APC^Δ468^ mice and we explored the fate of tumor infiltrating inflammatory cells. Paraffin sections of Swiss rolled small intestine and colon were stained with fluorescent antibodies against F4/80, Gr1 and CD4-FoxP3. Furthermore, we stained sequential sections with Chloro Acetate Esterase (CAE) for tumor infitrating mast cells. We analyzed 3 images of polyps sections from 5 mice each of the control group and the Zileuton group, by calculating the fluorescent cell fraction of the total cells visible in higher field.

**Fig 5:**
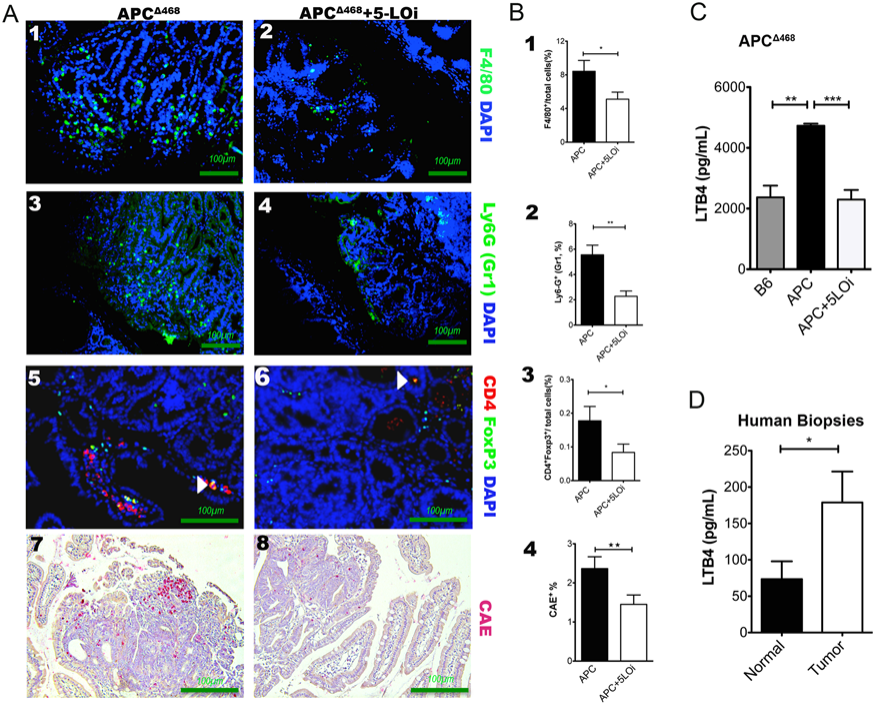
Zileuton treatment reduces the tumor infiltrating inflammatory cells in polyps. A) 1, 3, 5, 7: representative APC^Δ468/+^ polyps; 2, 4, 6, 8: representative Zileuton treated APC^Δ468/+^ polyps. B) calculation of positive cells as % frequency to the total cells; Black bars APC^Δ468/+,^ and open bars APC^Δ468/+^ Zileuton treated. C) LTB4 ELISA of B6 (gray bars), APC^Δ468/+^ (black bars), and APC^Δ468/+^ Zileuton treated (open bars). D) ELISA human biopsy extracts. C) LTB4 ELISA of B6 (gray bars), APC^Δ468/+^ (black bars), and APC^Δ468/+^ Zileuton treated (open bars). D) ELISA human biopsy extracts. Black bars healthy tissue, Open bars tumor extracts. one tailed unpaired t test with Welch’s correction, * P<0.05, **P<0.005, one tail unpaired t test.

In the Zileuton-treated polyps the frequency of F4/80^+^ cells was 50% lower compared to control polyps, the frequency Gr1^+^ infitrating cells was 60% lower than the control polyps and the infiltrating mast cells were 60% of the control. The marked reduction in the infitrating inflammatory cells in the Zileuton treated polyps might explain why only some polyps developed in these mice (Fig. 5A,B). In addition, NIRF endoscopy, which measures cathepsis activity *in vivo*, showed that Zileuton treatment decreased cathepsin activity in mouse polyps (S2 Fig.).

### 5-Lipoxygenase is elevated in human polyp extracts

The LTB4 concentration of the serum LTB4 was 50% lower in mice that were treated with Zileuton than in the untreated APC^Δ468^serum (Fig. 5C). To correlate our mouse findings with human disease, we colected human polyp tissues, as well as neighboring healthy tissue. Extracts of these tissues were analyzed with LTB4 ELISA. The mean concentration of LTB4 was more than 2-fold higher in the cancerous tissue extracts than the LTB4 concentation in the extracts of normal neighboring tissues (Fig. 5D).

## Discussion

Zileuton is an orally available and FDA approved drug for the treatment of patients with asthma, alleviating disease symptoms by suppressing 5-LO and LTB4 induced inflammation. In this study, we found that Zileuton suppressed LTB4 production in polyposis mice, resulting in reduced inflammation and consequently polyp burden. We also documented increased amounts of LTB4 in dysplastic tissues of patients with polyps and colon cancer. Previously Zileuton as used as anti-colitis agent in a rat experimental colitis rats [23] [24]. It is known that inflammation in colitis is pronounced and sustained because of loss of the protective epithelial layer of the lumen and exposure of the mucosa to the colonic bacteria. Consequently, LTB4 levels in these lesions are elevated. In dysplastic lesions the epithelial layer is intact and the inflammatory reactions originate from the development of these early lesions.

We have previously attempted to elucidate the effect of Zileuton treatment on colon cancer human epithelial cells [15]. and showed that established colon cancer cell lines have active 5-LO and produce the product LTB4 in vitro. Heterotopic injection in athymic mice developed very fast tumors since the immune system of these mice is compromised and these cells are highly mutated. Indeed Zileuton is reducing the growth of the injected epithelial cells. Heterotopic injection in athymic mice resulted in very rapid tumor growth since the immune system of these mice is compromised. Indeed Zileuton reduced the growth of the injected epithelial cells. The role of 5-LO in epithelial cancer cells growth was supported by the fact that 5-LO is up regulated in human polyps, and correlates with tumor grade and undifferentiated phenotype.

In the present article we report the increase of 5-LO activity in human polyps and cancer, by measuring the increase of the LTB4 product in the polyp tissue as compared to the neighboring healthy tissue.

Cancer-associated inflammation nurtures the tumor microenvironment in favor of tumor progression by providing fuel for epithelial cell growth and pro-angiogenic factors for vascularization and angiogenesis [25–28]. This inflammation consists of leukotriene tumor infiltrating producers: mast cells, basophils, eosinophils, neutrophils and macrophages. Mast cells are an essential hematopoietic component of polyp development [29]. Reduction of the frequency of intraepithelial MC with adaptive transfer resulted to a significant reduction of the numbers of polyps, as well as, their size. The MC frequency reduction is affecting the recruitment of tumor associated myeloid cells.

Through the production of 5-LO/LTB4, mast cells recruit MDSCs into the polyp, which in turn are capable of inhibiting protective anti-tumor T-cells [18,30]. LTB4 has also been shown to promote angiogenesis [31–33], and inhibition of 5-LO was able to reduce expression of VEGF and matrix metallo-proteases in colon cancer cells activated by cigarette smoke extract [34]. We detected decreased levels of VEGF after Zileuton treatment. Bevacizumab (Avastin) is an inhibitor of VEGF that was approved for the treatment of metastatic colon cancer patients in combination with chemotherapy after demonstrating enhanced patient survival compared to patients treated with chemotherapy alone [35,36]. Hence the use of Zileuton inhibition of 5-LO activity in polyposis or colon cancer could provide a dual benefit by suppressing both tumor-promoting inflammation and angiogenesis.

Inhibition of arachidonic acid metabolism via the COX pathway with NSAIDs or celecoxib has been tested in polyposis and colon cancer patients, and has shown some clinical benefit [37–40]. Celeoxib (Celebrex) was initially approved by the FDA to reduce the number of polyps in patients with familial adenomatous polyposis [41], but the indication has since been withdrawn. Serious cardiovascular and gastrointestinal adverse events are associated with long-term use of celecoxib and NSAIDs. However, given the observed clinical benefits in treated patients, finding a means to target the arachidonic acid pathway, while minimizing adverse events associated with the therapy is needed. Our studies support the idea that Zileuton could be a safe alternative; we showed that Zileuton was capable of suppressing polyposis, and the drug has been tested in thousands of patients with asthma exhibiting minimal side effects. Furthermore, Zileuton has also been shown to suppress prostaglandin biosynthesis, inhibiting the production of PGE2 [42]. PGE2 is a down-stream product of COX-2 activity [43]. Hence, an additional benefit with Zileuton treatment in polyposis and colon cancer patients could be dual suppression of prostaglandin biosynthesis and 5-LO resulting in decreased tumor growth.

Zileuton was tested in a randomized phase II trial in patients with advanced non-small cell lung cancer [41]. Patients received carboplatin/gemcitabine with Zileuton, celecoxib, or both Zileuton and celecoxib. The study failed to demonstrate a survival benefit in any arm, but the drugs, including Zileuton, were well tolerated. It is reasonable to suggest that the benefits of Zileuton might only be seen during escalation of cancer-associated inflammation and thus might have more use in cancer as a chemo-preventive agent, which is the setting in which we tested in this study.

Our findings indicate that 5-LO-specific inhibition may be a promising therapeutic strategy for the improvement of prognosis for patients with polyposis and in other patient populations at high risk for developing sporadic adenomatous polyps and colon cancer. The current availability of the FDA-approved 5-LO inhibitor Zileuton prompts further evaluation as a potential chemo-preventive agent.

## Supporting Information

S1 Fig Zileuton fed mice have increased weight and decreased spleen size. The APC^Δ468^ control group mice weighed 19.25±0.7 g versus 26.7±2.6 g for the APC^Δ468^ Zileuton, *P* = 0.0087, unpaired t test and their spleen was much smaller (463.9±62 mg for APC^Δ468^ control group mice vs 165.4±55.15 mg for the APC^Δ468^ Zileuton, *P* = 0.046, unpaired t test.
(TIF)

S2 Fig Zileuton treatment reduces the cathepsin activity mean fluorescence intensity of the polyps as detected *in vivo*. *A)* Representative images of a polyp as can be detected with NIRF endoscope using NIRF cathepsin activity probes; Red signal cathepsin activity. B) Bar graph of the MFI of the images from NIRF endoscopy and reflectence fluorescence. Black bars APC^Δ468/+,^ and open bars APC^Δ468/+^ Zileuton treated.
(TIF)
