# Supplementary figures and images for: Zileuton, 5-Lipoxygenase Inhibitor, Acts as a Chemopreventive Agent in Intestinal Polyposis, by Modulating Polyp and Systemic Inflammation
Source: PLoS One. 2015 Mar 6;10(3):e0121402. doi: 10.1371/journal.pone.0121402 (PMC4351892; doi:10.1371/journal.pone.0121402)

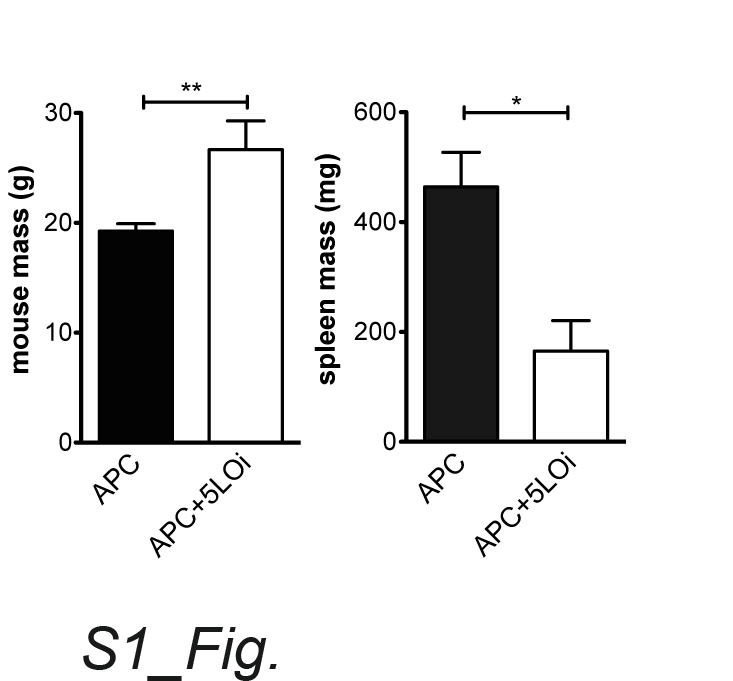

Supplement: S1 Fig — The APCΔ468 control group mice weighed 19.25±0.7 g versus 26.7±2.6 g for the APCΔ468 Zileuton, P = 0.0087, unpaired t test and their spleen was much smaller (463.9±62 mg for APCΔ468 control group mice vs 165.4±55.15 mg for the APCΔ468 Zileuton, P = 0.046, unpaired t test. (TIF) [file pone.0121402.s001.tif]

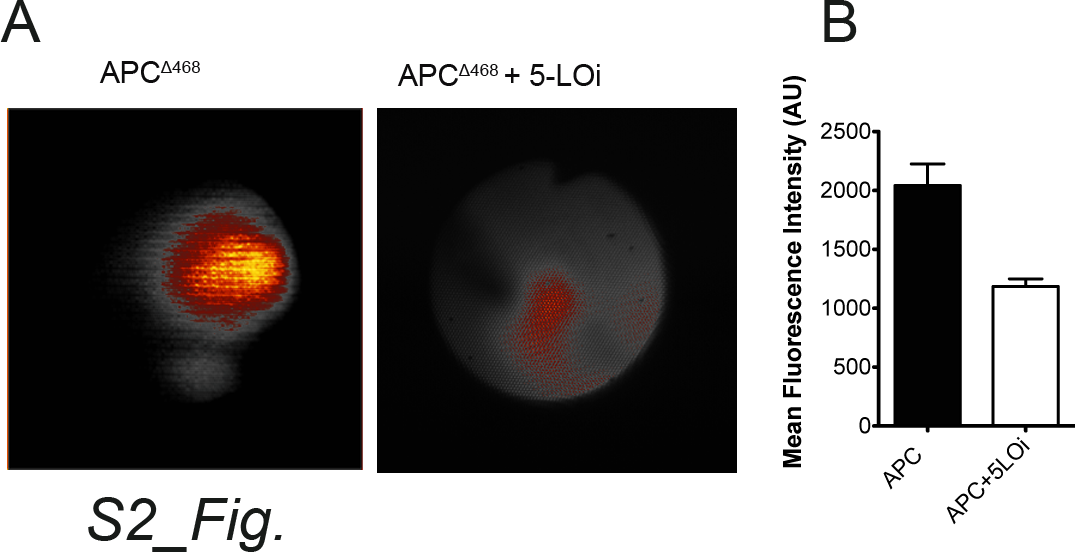

Supplement: S2 Fig — A) Representative images of a polyp as can be detected with NIRF endoscope using NIRF cathepsin activity probes; Red signal cathepsin activity. B) Bar graph of the MFI of the images from NIRF endoscopy and reflectence fluorescence. Black bars APCΔ468/+, and open bars APCΔ468/+ Zileuton treated. (TIF) [file pone.0121402.s002.tif]
